# Evaluating the Implementation of the Pediatric Acute Care Education (PACE) Program in Northwestern Tanzania: A Mixed-Methods Study Guided by Normalization Process Theory

**DOI:** 10.21203/rs.3.rs-4432440/v1

**Published:** 2024-05-31

**Authors:** Joseph R Mwanga, Adolfine Hokororo, Hanston Ndosi, Theopista Masenge, Florence S Kalabamu, Daniel Tawfik, Rishi P Mediratta, Boris Rozenfeld, Marc Berg, Zachary H Smith, Neema Chami, Namala P Mkopi, Castory Mwanga, Enock Diocles, Ambrose Agweyu, Peter A Meaney

**Affiliations:** Catholic University of Health and Allied Sciences; Catholic University of Health and Allied Sciences; Catholic University of Health and Allied Sciences; Pediatric Association of Tanzania; Pediatric Association of Tanzania; Stanford University School of Medicine; Stanford University School of Medicine; Area9 Lyceum; Stanford University School of Medicine; Kaiser Permanente; Catholic University of Health and Allied Sciences; Pediatric Association of Tanzania; Pediatric Association of Tanzania; Catholic University of Health and Allied Sciences; London School of Hygiene & Tropical Medicine; Stanford University School of Medicine

**Keywords:** Adaptive E-learning, Feasibility, Acceptability, Normalization Process Theory, Implementation Science, Pediatrics, Tanzania

## Abstract

**Background:**

In low- and -middle-income countries (LMICs) like Tanzania, the competency of healthcare providers critically influences the quality of pediatric care. To address this, we introduced PACE (Pediatric Acute Care Education), an adaptive e-learning program tailored to enhance provider competency in line with Tanzania’s national guidelines for managing seriously ill children. Adaptive e-learning presents a promising alternative to traditional in-service education, yet optimal strategies for its implementation in LMIC settings remain to be fully elucidated.

**Objectives:**

This study aimed to (1) evaluate the initial implementation of PACE in Mwanza, Tanzania, using the constructs of Normalization Process Theory (NPT), and (2) provide insights into its feasibility, acceptability, and scalability potential.

**Methods:**

A mixed-methods approach was employed across three healthcare settings in Mwanza: a zonal hospital and two health centers. NPT was utilized to navigate the complexities of implementing PACE. Data collection involved a customized NoMAD survey, focus groups and in-depth interviews with healthcare providers.

**Results:**

The study engaged 82 healthcare providers through the NoMAD survey and 79 in focus groups and interviews. Findings indicated high levels of coherence and cognitive participation, demonstrating that PACE is well-understood and resonates with existing healthcare goals. Providers expressed a willingness to integrate PACE into their practice, distinguishing it from existing educational methods. However, challenges related to resources and infrastructure, particularly affecting collective action, were noted. The short duration of the study limited the assessment of reflexive monitoring, though early indicators point towards the potential for PACE’s long-term sustainability.

**Conclusion:**

This study offers vital insights into the feasibility and acceptability of implementing PACE in a Tanzanian context. While PACE aligns well with healthcare objectives, addressing resource and infrastructure challenges is crucial for its successful and sustainable implementation. Furthermore, the study underscores the value of NPT as a framework in guiding implementation processes, with broader implications for implementation science and pediatric acute care in LMICs.

## Introduction to Results

The results section is organized to provide an in-depth analysis of both the implementation and impact of the Pediatric Acute Care Education (PACE) program. Initially, “Provider Demographics and Work Perceptions,” offers three layers of data: descriptive statistics that characterize the healthcare providers who took part in the NoMAD survey, comparative analyses that differentiate between providers from a zonal hospital and health centers, and emergent themes from in-depth interviews (IDIs) and focus group discussions (FGDs) that shed light on providers’ general perceptions of their roles in pediatric acute care. Following this, “Normalization Process Theory (NPT) Constructs” is bifurcated into an overview of general perceptions regarding PACE implementation and a detailed examination of the four principal NPT constructs—Coherence, Cognitive Participation, Collective Action, and Reflexive Monitoring. Each construct is scrutinized through a tripartite lens: descriptive data from the NoMAD survey, comparative analyses across different healthcare settings, and qualitative insights derived from IDIs and FGDs. Finally, “Summary of Feasibility, Acceptability, and Scalability,” synthesizes all findings into descriptive and comparative categories to offer a comprehensive evaluation of PACE’s potential for normalization within healthcare settings, while deliberately omitting thematic categories for a more focused interpretation.

### Provider Demographics and Work Perceptions

Eighty-two of 272 healthcare providers completed the NoMAD survey, yielding a response rate of 30%. Of the 82 healthcare providers: 59 were from the zonal hospital and 23 from health centers. Median ages were 27 and 29 years for the zonal hospital and health centers, respectively (**Table 2**). Gender distribution was similar in both settings (39% female in the zonal hospital, 43.5% in health centers). Significant cadre differences existed: the zonal hospital had more medical (47.5% vs. 8.7%) and nursing officers (42.4% vs. 30.4%), while health centers had more clinical officers (30.4% vs. 0%). Clinical experience varied, with medians of 1 year at the zonal hospital and 4 years at health centers (p-value: 0.004). Both settings had over 70% of providers with prior training. Job satisfaction scores were not Significantly different between the two groups.

### IDI and Focus Group demographics.

Seventy-nine healthcare providers participated in the study: 24 senior providers were interviewed (18 zonal hospital, 6 health centers) and 13 focus groups were held with junior providers (39 zonal hospital and 16 health centers). The focus group discussions varied in size, with an average of 4 participants per group. All cadres were represented: medical officers (26), nursing officers (19), interns (16), clinical officers (12), Assistant medical officers (3), and medical attendants (3). Clinical experience ranged from 1 to 20 years.

#### Focus group themes for Provider Demographics and Work Perceptions.

The overarching theme from the IDIs and FGDs was healthcare providers’ active engagement in pediatric care, particularly for seriously ill newborns and children. The quotes elucidate the providers’ daily responsibilities and specialized roles across different settings.

A provider noted, “I deal with ill children daily. There’s always a new transfer or admission.” This statement highlights the relentless nature of pediatric care, emphasizing the ongoing attention to both new and existing cases.

Another provider said, “I specialize in neonatology but attend to all children as per our country’s laws.” This underscores the specialized yet comprehensive roles some providers assume, adhering to broader healthcare mandates.

A provider added, “I work in the labour ward, treating newborns who fall ill before reaching twenty-eight days.” This comment reveals that even those in labour wards have pediatric responsibilities, extending the scope of care to include newborns who become ill.

In summary, the quotes collectively depict healthcare providers as deeply committed to pediatric care, each with specialized roles but universally focused on patient well-being.

### NoMAD General Items

**Figure 1:**
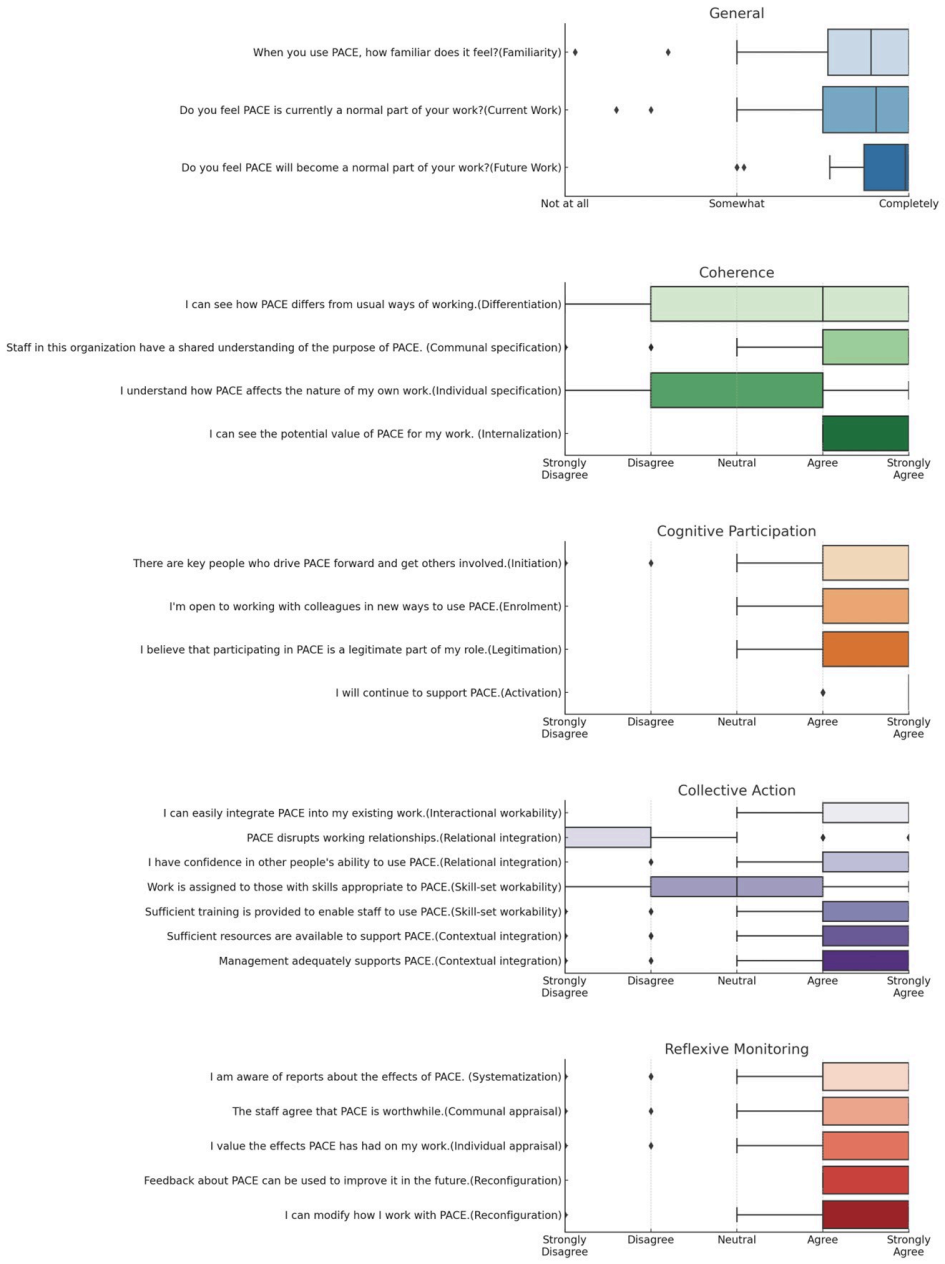
Boxplot of Participant Responses to NoMAD Survey by NPT construct and subconstruct.

Respondents showed high familiarity with PACE, evidenced by a median score of 89 out of 100, although an interquartile range of 76–100 indicates variability (Table 3, Fig. 1). General satisfaction with PACE in current work had a median of 91 and a similar opinion range, as suggested by an interquartile range of 75–100. Optimism for PACE in future work was highest, with a median of 99 and an interquartile range of 87–100. No Significant differences were observed between the zonal hospital and health centers in these variables.

### Normalization Process Theory (NPT) Constructs Coherence

#### NoMAD data analysis

Coherence, a key construct in PACE implementation, and its subconstructs reveal important trends. “Communal Specification” and “Internalization” had median scores of 2 (Agree) and 1 (Strongly Agree), respectively, with interquartile ranges of 1–2 for both indicating strong collective agreement on PACE’s purpose and value (**Table 3**, Fig. 1). “Differentiation” and “Individual Specification” both had medians of 2 (Agree), suggesting moderate agreement that PACE is distinct, and roles are understood. Interquartile ranges were 1–4 for “Differentiation” and 2–4 for “Individual Specification,” indicating variability within subconstructs. Overall, data suggest strong collective and individual agreement on PACE’s differentiation and value.

For Coherence subconstructs, there were no Significant differences in median scores or IQRs between settings indicating uniform perceptions of purpose, distinctiveness, and valuation of PACE across settings.

##### Coherence Themes from IDI and FGD.

###### Differentiation:

Providers value PACE for its detailed guidance on specific pediatric cases, such as difficulty breathing, which was not covered in their basic training. One provider said, “PACE goes beyond basic knowledge, offering detailed steps for managing cases like difficulty breathing. This is a Significant advantage.”(**Table 4**)

###### Communal Specification:

PACE is seen as a tool for empowering providers to reduce child mortality and improve service quality, aligning with facility goals. One provider remarked, “PACE aims to empower us to reduce child mortality,” while another noted, “Its objectives align with our hospital’s goals to update healthcare providers’ knowledge.”

###### Individual Specification:

Providers believe PACE has enhanced their understanding and management of seriously ill children. One provider observed, “Before PACE, I relied on existing procedures and guidelines. Now, I’ve gained new insights that could positively impact our treatment system.”

###### Internalization:

Providers find that PACE is consistent with Tanzanian and WHO guidelines, useful both in their work and in training medical students. One provider said, “PACE refreshes my memory and aligns with existing guidelines. I use it to educate medical students and junior doctors.”

### Cognitive participation

#### NoMAD data analysis

Cognitive Participation, a construct examining collaboration around PACE, reveals strong agreement across its subconstructs. “Activation,” measuring ongoing PACE support, had a median of 1, indicating strong agreement (**Table 3**, Fig. 1). “Enrolment” and “Initiation,” with medians of 1 for both, also showed strong agreement for participation and leadership in PACE. “Legitimation,” assessing PACE’s work integration, also had a median of 1, suggesting strong agreement. IQRs ranged from 1–1 to 1–2, indicating some dispersion within subconstructs. Overall, data suggest strong agreement on PACE’s support, participation, leadership, and legitimacy.

There were no Significant differences by setting, indicating consistent strong agreement on PACE across settings.

##### Cognitive Participation Themes from IDI and FGD.

###### Initiation:

Providers were introduced to PACE by colleagues and supervisors, prompting them to enroll. One provider said, “Specialists introduced us to PACE, and we started learning.” Another noted, “After seeing a colleague engage with PACE, I joined too.” Some providers find individual initiation beneficial, as one stated, “Starting alone is effective.” (**Table 4**)

###### Legitimation:

PACE is seen as empowering providers to enhance their pediatric care. One provider noted, “PACE taught me how to administer oxygen based on a child’s age.” Another highlighted PACE’s flexibility, saying, “You can engage with PACE individually or in groups.”

###### Enrolment:

Providers mainly use PACE individually but also share modules to spread knowledge. One provider said, “I often use PACE on my phone but also share modules with colleagues.”

###### Activation:

Despite busy schedules, providers are committed to PACE training. One provider stated, “Our commitment helps translate knowledge into practice.” Another emphasized their personal dedication, saying, “I find time to study PACE multiple times a week, showing my commitment.”

### Collective Action

#### NoMAD data analysis

In the realm of Collective Action, focusing on collaborative PACE enactment, subconstructs reveal nuanced insights. “Interactional Workability,” with a median of 1, suggests work required by PACE is generally manageable (**Table 3**, Fig. 1). “Relational Integration” showed strong disagreement that PACE disrupts relationships but agreement that it can be effectively used by peers. “Skill-set Workability” indicated mixed alignment with provider needs but strong consensus on sufficient PACE training. “Contextual Integration” had medians of 2, suggesting strong organizational support. Overall, data suggest varying agreement levels on work manageability, interpersonal relations, task allocation, and organizational support.

“Relational Integration” showed a Significant difference in one subconstruct (p = 0.02), suggesting higher trust levels in the zonal hospital. There were no other differences by setting. Overall, data suggest consistent Collective Action approaches across settings, with a trust advantage in the zonal hospital.

##### Collective Action Themes from IDI and FGD.

###### Interactional Workability:

PACE’s digital format allows for individual study and facilitates group discussions. One provider noted, “PACE’s digital nature allows for flexible study schedules.” Group discussions often occur in the mornings, as another provider said, “We discuss PACE modules early before attending to patients.” (**Table 4**)

###### Relational Integration:

Initially, providers engaged with PACE for personal benefit but later saw the value in sharing knowledge. One provider stated, “I initially used PACE for personal growth but later realized the importance of sharing this knowledge.” Teamwork and collective benefits were emphasized, with one provider noting, “We work as a team to meet our objectives.”

###### Skill-set Workability:

Providers value the practical application of PACE knowledge in patient care. One provider said, “After learning, it’s crucial to apply this knowledge in treating patients.”

###### Contextual Integration:

Challenges like inadequate supplies and lack of electricity hinder PACE implementation. One provider stated, ““Sometimes we face difficulties such as inadequate supply of medical equipment and supplies. For example, there is a child in need of oxygen while there is no electricity and we do not have standby generator. This becomes a barrier to translating PACE in practice.” However, the availability of tools and support from PACE management facilitates implementation, as another provider noted, “Availability of tools and support has eased PACE’s translation into practice.”

### Reflexive Monitoring

#### NoMAD data analysis

In the context of Reflexive Monitoring, focusing on PACE appraisal, subconstructs reveal varying agreement levels. “Communal Appraisal” had a median of 2, suggesting strong collective agreement on PACE’s benefit (**Table 3**, Fig. 1). “Individual Appraisal” had a median of 1, indicating strong individual agreement. “Reconfiguration,” examining work modifications due to PACE, had a median of 2, showing slight agreement for work adjustments. “Systematization,” assessing information access on PACE effects, had a median of 2, leaning towards strong agreement. Overall, data suggest strong agreement on PACE’s collective and individual appraisal, work modification, and information access.

There were no Significant differences by setting, suggesting consistent agreement on PACE appraisal and modification across settings.

##### Reflexive Monitoring Themes from IDI and FGD.

###### Systematization:

No quotes directly address this subconstruct, suggesting a need for further exploration within the PACE context.

###### Communal Appraisal:

Providers find PACE valuable for educating junior doctors, simplifying complex topics, and boosting confidence. One provider noted, “PACE aids in teaching junior doctors by simplifying complex topics and enhancing my confidence during discussions.” (Table 4)

###### Individual Appraisal:

Providers believe PACE has enriched their knowledge and confidence in pediatric care. Quotes summarizing this sentiment include, “I’ve gained confidence and can act quickly in emergencies,” and “I can provide timely service with increased courage.”

###### Reconfiguration:

A notable challenge is the inaccessibility of learned material for future reference, hindering providers’ ability to refresh their knowledge. One provider stated, “Once you complete a module, it becomes inaccessible, making it difficult to revisit for future case management.”

##### Summary of Feasibility, Acceptability, and Scalability.

Overall, PACE is generally feasible across healthcare settings, with providers across settings either agreeing or strongly agreeing that people do the work required by interventions and their components (Interactional Workability), the work of interventions and their components supported by host organizations (Contextual Integration).

PACE is also generally acceptable among healthcare providers. Providers collectively agree about the purpose of PACE and its components (Communal Specification), agree that PACE and its components are the right thing to do and should be part of their work (Legitimation), and the collectively and individually agree that PACE is worthwhile (Communal and Individual Appraisal).

Lastly, PACE appears to be scalable, with some variability in its adaptability and skill-set alignment. The scalability subconstructs, mapped to NPT, indicate a mean score of 1.23 and a standard deviation of 0.42 for Providers strongly agree that they will continue to support PACE and its components (Activation), that they can modify their work in response to their appraisal of PACE, and feedback can be used to improve it in the future (Reconfiguration). Providers agree or are neutral about the work of PACE and its components being appropriately allocated to people (Skill-set Workability), indicating more work is needed to identify the correct providers to participate in PACE or more support needs to be allocated to those providers to complete PACE.

### Summary of Main Results

#### Provider Demographics

- 82 healthcare providers participated in the NoMAD survey and 79 in interviews and discussions, with over 2/3 from the zonal hospital.
- Profession and years of clinical experience varied between zonal hospital and health centers with more physicians at the zonal hospital and more experienced providers at health centers.
- Healthcare providers are deeply involved in pediatric care and find value in PACE.

### NPT Constructs

#### General

- High levels of familiarity and positive outlook towards the PACE intervention.
- No Significant differences between the zonal hospital and health centers in general perceptions of PACE.

#### Coherence

- PACE is seen as aligning with facility goals.
- General agreement on the collective and individual understanding of PACE.
- No Significant differences between settings in understanding and planning for PACE.

#### Cognitive Participation

- Strong agreement on ongoing support, participation, and legitimacy of PACE.
- PACE is seen as beneficial for managing specific pediatric cases.
- Consistent levels of agreement across both zonal hospitals and health centers.

#### Collective Action

- Varying degrees of agreement on workability, interpersonal confidence, and organizational support.
- Generally consistent approach across both settings, with a notable difference in relational integration favoring the zonal hospital.
- Challenges: Inadequate supplies and lack of electricity noted as barriers to PACE implementation.

#### Reflexive Monitoring

- Strong agreement in the collective and individual appraisal of PACE.
- Consistent agreement across both settings in the appraisal and modification of PACE.
- Challenge: Inaccessibility of learned material for future reference.

#### Feasibility, Acceptability, Scalability

- PACE is generally feasible, acceptable, and potentially scalable across different healthcare settings, with some variability due to challenges and material inaccessibility.

## Background

Context and importance of the study. Healthcare providers’ in-service education in low-and-middle income countries (LMICs) is limited in reach, effectiveness and sustainability, and these limitations contribute to millions of child deaths each year.(1,2) Pneumonia, birth asphyxia, dehydration, malaria, malnutrition, and anaemia collectively cause over 4 million under-five deaths each year, half of those deaths occur in sub-Saharan Africa; and thousands of those deaths occur in Tanzania.(3,4) The government of Tanzania is committed to reducing neonatal mortality from 20 to the sustainable development goals (SDGs) target of 12/100,000 by the year 2030.(5)

Brief review of the literature. Provider knowledge and competency are two major drivers of care quality. (2,6) Unfortunately, conventional in-service education methods are often inadequate in coverage and difficult to sustain.(6) Conventional education methods do not systematically adapt to individual providers’ knowledge or convenience,(7,8) target minimal competency, and do not provide long-term increases in knowledge, which limits education effectiveness.(9,10)

Adaptive e-learning, characterized by its use of advanced technology such as artificial intelligence and data analytics, offers a promising solution to the limitations of traditional educational methods. This approach tailors the learning experience to each individual by dynamically adjusting content and instructional strategies based on the learner’s unique needs, abilities, and progress. Such personalization not only addresses the challenges of manpower and training resource shortages prevalent in low-and-middle-income countries (LMICs) but also represents a strategic innovation in disseminating knowledge effectively.

The World Health Organization (WHO) has underscored the importance of implementing e-learning solutions for healthcare workers globally.(11) Adaptive e-learning, with its capacity to adjust to individual learner needs, holds considerable promise for enhancing the efficiency of training healthcare workers. However, formal studies exploring the use of adaptive e-learning in LMIC contexts are scarce. Identifying and establishing best practices in e-learning and adaptive methodologies presents a Significant opportunity to enhance the dissemination and implementation of evidence-based interventions. Such advancements are crucial for improving the quality of care in these regions.

To address the existing limitations of current healthcare workers’ education in LMICs, we developed pediatric acute care education (PACE), an adaptive e-learning program focused on pneumonia, birth asphyxia, dehydration, malaria, malnutrition, and anaemia, and Tanzania’s national guidelines for the management of seriously-ill children as source material.(12,13) Prior to large-scale implementation, we undertook a feasibility trial of this curriculum among a cohort of medical interns at a zonal hospital in Tanzania. Then we continued to enroll healthcare providers in 8 health facilities under the Pediatric Association of Tanzania’s Clinical Learning Network facilities in Nyamagana and Ilemela districts of Mwanza region. This research report covers a qualitative pilot study that was conducted in three facilities to explore the feasibility and acceptability PACE.

Study aims and objectives. The primary aim of this research is to assess the preliminary implementation of the Pediatric Acute Care E-learning (PACE) intervention across two distinct types of pediatric acute care facilities: zonal hospitals and health centers. The study employs the Normalization Process Theory (NPT) framework in a twofold manner: first, using a tailored NoMAD survey instrument to evaluate the integration of PACE into routine clinical practice; and second, via in-depth interviews and focus group discussions to gain qualitative insights. These dual approaches aim to achieve two principal objectives. The first objective is to utilize the constructs and subconstructs of NPT as evaluative metrics for scrutinizing PACE’s implementation. The second objective is to consolidate these findings to provide a comprehensive analysis of the feasibility, acceptability, and scalability of the PACE intervention across the targeted healthcare settings.

## Methods

### Study Design

This study employed a mixed-methods approach to evaluate the implementation of the Pediatric Acute Care Education (PACE) program in healthcare settings in Northwestern Tanzania. We administered a tailored NoMAD survey post-intervention to healthcare providers in a zonal hospital and two health centers. Additionally, in-depth interviews and focus group discussions were conducted post-intervention to enrich the survey data. The study was guided by two primary objectives:

Objective 1: To use Normalization Process Theory (NPT) to assess the initial implementation of PACE.

Objective 2: To summarize the findings in terms of feasibility, acceptability, and scalability of PACE.

### Theoretical Framework

Normalization Process Theory (NPT) has been described as a sociological toolkit to help understand the dynamics of implementing, embedding, and integrating new technology, or complex intervention into routine practice.(14) NPT provides a conceptual framework for understanding and evaluating the processes (implementation) by which new health technologies and other complex interventions are routinely operationalized in everyday work (embedding), and sustained in practice (integration).(15–20) The theory is organized around four main constructs, each of which has its own subconstructs. (15) These constructs collectively offer insights into the feasibility, acceptability, and scalability of an intervention or innovation (Figure 1). Each of these constructs and subconstructs offers a unique lens through which the feasibility, acceptability, and scalability of a new practice can be evaluated, thereby aiding in its effective implementation.

### Study setting

The study was conducted between August 2022 and July 2023 at three healthcare facilities in Mwanza, Tanzania: Bugando Medical Centre (BMC), a zonal referral and teaching hospital; Makongoro Health Centre, located in the city center; and Igoma Health Centre, situated a few kilometers from the city center. All three facilities offer newborn and pediatric care among other health services.

### Providers

Eligibility criteria. Providers included physicians (specialist/superspecialist), nursing officers, medical officers, clinical officers, assistant medical officers, medical attendants, or other providers enrolled in PACE or senior facility staff that supervise PACE providers.

Recruitment process. Healthcare providers were informed about the study through their facility leaders, and individuals who responded to the survey were not necessarily the same as those who participated in the focus groups or in-depth interviews.

### Data Collection Tools

NoMAD questionnaire. The NoMAD is a 23-item questionnaire based on NPT, designed to assess the social processes influencing the integration of complex interventions.(18,21) It includes 3 general items, and 20 related to specific NPT constructs (4 Coherence, 7 Collective Action, 4 Cognitive Participation, 5 Reflexive Monitoring).The general items were on a scale of 0–100 and NPT construct items were modified to include a five-point Likert scale (1-Strongly Agree, 5-Strongly Disagree)and additional options for respondents to indicate if a question is not relevant to their role, stage, or the intervention itself. NPT subconstruct survey items are listed in **Table 1**, with the complete survey in the **Supplementary Materials.**

In-depth interviews (IDIs) and focus group discussions (FGDs). Interview guides were developed based on the previous experience with similar data collection tools. Training and pretesting of tools were conducted by study investigators.

### Data collection process.

NoMAD Survey. All PACE participants received the NoMAD survey invitations via WhatsApp 30 days post-intervention or upon initial learning completion of PACE. Data were collected through REDCap.

Focus Group Discussions and In-depth interviews. We employed a purposeful sampling strategy for the qualitative components, selecting senior healthcare providers for in-depth interviews and junior providers for focus group discussions (FGDs). This approach ensured that participants and sites provided valuable insights into the research problem and central phenomenon. The data collection comprised of 24 interviews and 13 FGDs, commenced with a series of field visits and was guided by Normalization Process Theory (NPT) constructs. This ensured thematic consistency across both methods and facilitated methodological triangulation. The focus groups, segregated by sex but including a mix of cadres from each health facility, enriched the diversity of perspectives. The iterative nature of our methodology allowed for continuous refinement of our theoretical framework, methodologies, and sampling strategies, informed by emerging data. Consequently, the guides for both interviews and FGDs were dynamically modified to reflect the evolving study themes. All sessions were conducted in Kiswahili at the providers’ work premises, adding contextual depth, and were meticulously audio-recorded, transcribed verbatim, and translated into English for analysis.

### Data Analysis

Quantitative Analysis: Descriptive statistics are reported as frequencies and percentages or medians and interquartile ranges, with comparisons via Fisher’s exact test or Mann-Whitney U test as appropriate. Analyses were conducted using Stata 17.0 (Stata Corp, College Station, TX, USA).

Qualitative Analysis: The analysis process, conducted concurrently with data collection, was instrumental in achieving theoretical saturation, marked by the cessation of new information from ongoing interviews and FGDs. To ensure the validity and depth of our findings, we implemented member checking and investigator triangulation, with two independent investigators coding and interpreting the data using NVivo 2020 software (QSR International Pty Ltd., Sydney, Australia). This software facilitated a hybrid coding approach that blended deductive and inductive methods for a comprehensive thematic content analysis. Contextual insights from the interviews and discussions were key to interpreting the findings, with representative quotations included to illustrate identified themes. Data triangulation was achieved using diverse data sources, and the research team’s expertise further enhanced the rigor and reflexivity of the analysis.

Summarizing for feasibility acceptability and scalability. We used Proctors definition of implementation outcomes and mapped to NPT subconstructs using the definition by May et al.(22,23)

Feasibility is concerned with the practical aspects of implementing a new intervention, including resource allocation, training, and ease of integration into existing work. In NPT, this aligns closely with the construct of “Collective Action,” which refers to the operational work that people do to enact a set of practices. To assess feasibility, we interpreted responses to “Sufficient training is provided to enable staff to use PACE” (collective action, skill set workability); “Sufficient resources are available to support PACE” and “Management adequately supports PACE” (collective action, contextual integration); and “I can easily integrate PACE into my existing work” (collective action, interactional workability).

Acceptability refers to the extent to which the new intervention is agreeable or satisfactory among its users. To assess acceptability, we interpreted responses to “Staff in this organization have a shared understanding of the purpose of PACE” (coherence: communal Specification); “I believe that participating in PACE is a legitimate part of my role” (cognitive participation, legitimation); “The staff agree that PACE is worthwhile” (reflexive monitoring, communal appraisal); and “I value the effects PACE has had on my work” (reflexive monitoring, individual appraisal). In addition, we will compare scores between the zonal hospital and health centers.

Scalability involves the ability to expand the intervention to other settings while maintaining its effectiveness. To assess scalability, we interpreted responses to “I will continue to support PACE” (cognitive participation, activation); “Work is assigned to those with skills appropriate to PACE” (collective action, skill set workability); Feedback about PACE can be used to improve it in the future” and “I can modify how I work with PACE” (reflexive monitoring, reconfiguration).

Ethical Considerations. All providers provided informed consent, and the study was approved by the relevant ethical review boards.

Techniques to enhance trustworthiness. Since processing and analysis of qualitative data was systematic, explicit, and reproducible, the validation and trustworthiness of the findings was established. (24)

Reporting Guidelines. This study adheres to the STROBE and SRQR reporting guidelines for comprehensive and explicit reporting of observational and qualitative studies, respectively.(25,26)

## Results

### Introduction to Results

The results section is organized to provide an in-depth analysis of both the implementation and impact of the Pediatric Acute Care Education (PACE) program. Initially, “Provider Demographics and Work Perceptions,” offers three layers of data: descriptive statistics that characterize the healthcare providers who took part in the NoMAD survey, comparative analyses that differentiate between providers from a zonal hospital and health centers, and emergent themes from in-depth interviews (IDIs) and focus group discussions (FGDs) that shed light on providers’ general perceptions of their roles in pediatric acute care. Following this, “Normalization Process Theory (NPT) Constructs” is bifurcated into an overview of general perceptions regarding PACE implementation and a detailed examination of the four principal NPT constructs—Coherence, Cognitive Participation, Collective Action, and Reflexive Monitoring. Each construct is scrutinized through a tripartite lens: descriptive data from the NoMAD survey, comparative analyses across different healthcare settings, and qualitative insights derived from IDIs and FGDs. Finally, “Summary of Feasibility, Acceptability, and Scalability,” synthesizes all findings into descriptive and comparative categories to offer a comprehensive evaluation of PACE’s potential for normalization within healthcare settings, while deliberately omitting thematic categories for a more focused interpretation.

### Provider Demographics and Work Perceptions

Eighty-two of 272 healthcare providers completed the NoMAD survey, yielding a response rate of 30%. Of the 82 healthcare providers: 59 were from the zonal hospital and 23 from health centers. Median ages were 27 and 29 years for the zonal hospital and health centers, respectively (Table 2). Gender distribution was similar in both settings (39% female in the zonal hospital, 43.5% in health centers). Significant cadre differences existed: the zonal hospital had more medical (47.5% vs. 8.7%) and nursing officers (42.4% vs. 30.4%), while health centers had more clinical officers (30.4% vs. 0%). Clinical experience varied, with medians of 1 year at the zonal hospital and 4 years at health centers (p-value: 0.004). Both settings had over 70% of providers with prior training. Job satisfaction scores were not Significantly different between the two groups.

### IDI and Focus Group demographics.

Seventy-nine healthcare providers participated in the study: 24 senior providers were interviewed (18 zonal hospital, 6 health centers) and 13 focus groups were held with junior providers (39 zonal hospital and 16 health centers). The focus group discussions varied in size, with an average of 4 participants per group. All cadres were represented: medical officers (26), nursing officers (19), interns (16), clinical officers (12), Assistant medical officers (3), and medical attendants (3). Clinical experience ranged from 1 to 20 years.

### Focus group themes for Provider Demographics and Work Perceptions.

The overarching theme from the IDIs and FGDs was healthcare providers’ active engagement in pediatric care, particularly for seriously ill newborns and children. The quotes elucidate the providers’ daily responsibilities and specialized roles across different settings.

A provider noted, “I deal with ill children daily. There’s always a new transfer or admission.” This statement highlights the relentless nature of pediatric care, emphasizing the ongoing attention to both new and existing cases.

Another provider said, “I specialize in neonatology but attend to all children as per our country’s laws.” This underscores the specialized yet comprehensive roles some providers assume, adhering to broader healthcare mandates.

A provider added, “I work in the labour ward, treating newborns who fall ill before reaching twenty-eight days.” This comment reveals that even those in labour wards have pediatric responsibilities, extending the scope of care to include newborns who become ill.

In summary, the quotes collectively depict healthcare providers as deeply committed to pediatric care, each with specialized roles but universally focused on patient well-being.

### NoMAD General Items

Respondents showed high familiarity with PACE, evidenced by a median score of 89 out of 100, although an interquartile range of 76–100 indicates variability (Table 3, Figure 1). General satisfaction with PACE in current work had a median of 91 and a similar opinion range, as suggested by an interquartile range of 75–100. Optimism for PACE in future work was highest, with a median of 99 and an interquartile range of 87–100. No Significant differences were observed between the zonal hospital and health centers in these variables.

### Normalization Process Theory (NPT) Constructs

#### Coherence

##### NoMAD data analysis

Coherence, a key construct in PACE implementation, and its subconstructs reveal important trends. “Communal Specification” and “Internalization” had median scores of 2 (Agree) and 1 (Strongly Agree), respectively, with interquartile ranges of 1–2 for both indicating strong collective agreement on PACE’s purpose and value (**Table 3, Figure 1**). “Differentiation” and “Individual Specification” both had medians of 2 (Agree), suggesting moderate agreement that PACE is distinct, and roles are understood. Interquartile ranges were 1–4 for “Differentiation” and 2–4 for “Individual Specification,” indicating variability within subconstructs. Overall, data suggest strong collective and individual agreement on PACE’s differentiation and value.

For Coherence subconstructs, there were no Significant differences in median scores or IQRs between settings indicating uniform perceptions of purpose, distinctiveness, and valuation of PACE across settings.

##### Coherence Themes from IDI and FGD.

Differentiation: Providers value PACE for its detailed guidance on specific pediatric cases, such as difficulty breathing, which was not covered in their basic training. One provider said, “PACE goes beyond basic knowledge, offering detailed steps for managing cases like difficulty breathing. This is a Significant advantage.”(**Table 4**)

Communal Specification: PACE is seen as a tool for empowering providers to reduce child mortality and improve service quality, aligning with facility goals. One provider remarked, “PACE aims to empower us to reduce child mortality,” while another noted, “Its objectives align with our hospital’s goals to update healthcare providers’ knowledge.”

Individual Specification: Providers believe PACE has enhanced their understanding and management of seriously ill children. One provider observed, “Before PACE, I relied on existing procedures and guidelines. Now, I’ve gained new insights that could positively impact our treatment system.”

Internalization: Providers find that PACE is consistent with Tanzanian and WHO guidelines, useful both in their work and in training medical students. One provider said, “PACE refreshes my memory and aligns with existing guidelines. I use it to educate medical students and junior doctors.”

### Cognitive participation

#### NoMAD data analysis

Cognitive Participation, a construct examining collaboration around PACE, reveals strong agreement across its subconstructs. “Activation,” measuring ongoing PACE support, had a median of 1, indicating strong agreement (**Table 3**, Figure 1). “Enrolment” and “Initiation,” with medians of 1 for both, also showed strong agreement for participation and leadership in PACE. “Legitimation,” assessing PACE’s work integration, also had a median of 1, suggesting strong agreement. IQRs ranged from 1–1 to 1–2, indicating some dispersion within subconstructs. Overall, data suggest strong agreement on PACE’s support, participation, leadership, and legitimacy.

There were no Significant differences by setting, indicating consistent strong agreement on PACE across settings.

##### Cognitive Participation Themes from IDI and FGD.

###### Initiation:

Providers were introduced to PACE by colleagues and supervisors, prompting them to enroll. One provider said, “Specialists introduced us to PACE, and we started learning.” Another noted, “After seeing a colleague engage with PACE, I joined too.” Some providers find individual initiation beneficial, as one stated, “Starting alone is effective.” (**Table 4**)

###### Legitimation:

PACE is seen as empowering providers to enhance their pediatric care. One provider noted, “PACE taught me how to administer oxygen based on a child’s age.” Another highlighted PACE’s flexibility, saying, “You can engage with PACE individually or in groups.”

###### Enrolment:

Providers mainly use PACE individually but also share modules to spread knowledge. One provider said, “I often use PACE on my phone but also share modules with colleagues.”

###### Activation:

Despite busy schedules, providers are committed to PACE training. One provider stated, “Our commitment helps translate knowledge into practice.” Another emphasized their personal dedication, saying, “I find time to study PACE multiple times a week, showing my commitment.”

### Collective Action

#### NoMAD data analysis

In the realm of Collective Action, focusing on collaborative PACE enactment, subconstructs reveal nuanced insights. “Interactional Workability,” with a median of 1, suggests work required by PACE is generally manageable (**Table 3**, Figure 1). “Relational Integration” showed strong disagreement that PACE disrupts relationships but agreement that it can be effectively used by peers. “Skill-set Workability” indicated mixed alignment with provider needs but strong consensus on sufficient PACE training. “Contextual Integration” had medians of 2, suggesting strong organizational support. Overall, data suggest varying agreement levels on work manageability, interpersonal relations, task allocation, and organizational support.

“Relational Integration” showed a Significant difference in one subconstruct (p=0.02), suggesting higher trust levels in the zonal hospital. There were no other differences by setting. Overall, data suggest consistent Collective Action approaches across settings, with a trust advantage in the zonal hospital.

##### Collective Action Themes from IDI and FGD.

###### Interactional Workability:

PACE’s digital format allows for individual study and facilitates group discussions. One provider noted, “PACE’s digital nature allows for flexible study schedules.” Group discussions often occur in the mornings, as another provider said, “We discuss PACE modules early before attending to patients.” (**Table 4**)

###### Relational Integration:

Initially, providers engaged with PACE for personal benefit but later saw the value in sharing knowledge. One provider stated, “I initially used PACE for personal growth but later realized the importance of sharing this knowledge.” Teamwork and collective benefits were emphasized, with one provider noting, “We work as a team to meet our objectives.”

###### Skill-set Workability:

Providers value the practical application of PACE knowledge in patient care. One provider said, “After learning, it’s crucial to apply this knowledge in treating patients.”

###### Contextual Integration:

Challenges like inadequate supplies and lack of electricity hinder PACE implementation. One provider stated, ““Sometimes we face difficulties such as inadequate supply of medical equipment and supplies. For example, there is a child in need of oxygen while there is no electricity and we do not have standby generator. This becomes a barrier to translating PACE in practice.” However, the availability of tools and support from PACE management facilitates implementation, as another provider noted, “Availability of tools and support has eased PACE’s translation into practice.”

### Reflexive Monitoring

#### NoMAD data analysis

In the context of Reflexive Monitoring, focusing on PACE appraisal, subconstructs reveal varying agreement levels. “Communal Appraisal” had a median of 2, suggesting strong collective agreement on PACE’s benefit (**Table 3, Figure 1**). “Individual Appraisal” had a median of 1, indicating strong individual agreement. “Reconfiguration,” examining work modifications due to PACE, had a median of 2, showing slight agreement for work adjustments. “Systematization,” assessing information access on PACE effects, had a median of 2, leaning towards strong agreement. Overall, data suggest strong agreement on PACE’s collective and individual appraisal, work modification, and information access.

There were no Significant differences by setting, suggesting consistent agreement on PACE appraisal and modification across settings.

##### Reflexive Monitoring Themes from IDI and FGD.

Systematization: No quotes directly address this subconstruct, suggesting a need for further exploration within the PACE context.

Communal Appraisal: Providers find PACE valuable for educating junior doctors, simplifying complex topics, and boosting confidence. One provider noted, “PACE aids in teaching junior doctors by simplifying complex topics and enhancing my confidence during discussions.” (**Table 4**)

Individual Appraisal: Providers believe PACE has enriched their knowledge and confidence in pediatric care. Quotes summarizing this sentiment include, “I’ve gained confidence and can act quickly in emergencies,” and “I can provide timely service with increased courage.”

Reconfiguration: A notable challenge is the inaccessibility of learned material for future reference, hindering providers’ ability to refresh their knowledge. One provider stated, “Once you complete a module, it becomes inaccessible, making it difficult to revisit for future case management.”

### Summary of Feasibility, Acceptability, and Scalability.

Overall, PACE is generally feasible across healthcare settings, with providers across settings either agreeing or strongly agreeing that people do the work required by interventions and their components (Interactional Workability), the work of interventions and their components supported by host organizations (Contextual Integration).

PACE is also generally acceptable among healthcare providers. Providers collectively agree about the purpose of PACE and its components (Communal Specification), agree that PACE and its components are the right thing to do and should be part of their work (Legitimation), and the collectively and individually agree that PACE is worthwhile (Communal and Individual Appraisal).

Lastly, PACE appears to be scalable, with some variability in its adaptability and skill-set alignment. The scalability subconstructs, mapped to NPT, indicate a mean score of 1.23 and a standard deviation of 0.42 for Providers strongly agree that they will continue to support PACE and its components (Activation), that they can modify their work in response to their appraisal of PACE, and feedback can be used to improve it in the future (Reconfiguration). Providers agree or are neutral about the work of PACE and its components being appropriately allocated to people (Skill-set Workability), indicating more work is needed to identify the correct providers to participate in PACE or more support needs to be allocated to those providers to complete PACE.

### Summary of Main Results

#### Provider Demographics

- 82 healthcare providers participated in the NoMAD survey and 79 in interviews and discussions, with over 2/3 from the zonal hospital.
- Profession and years of clinical experience varied between zonal hospital and health centers with more physicians at the zonal hospital and more experienced providers at health centers.
- Healthcare providers are deeply involved in pediatric care and find value in PACE.

### NPT Constructs

#### General

- High levels of familiarity and positive outlook towards the PACE intervention.
- No Significant differences between the zonal hospital and health centers in general perceptions of PACE.

#### Coherence

- PACE is seen as aligning with facility goals.
- General agreement on the collective and individual understanding of PACE.
- No Significant differences between settings in understanding and planning for PACE.

#### Cognitive Participation

- Strong agreement on ongoing support, participation, and legitimacy of PACE.
- PACE is seen as beneficial for managing specific pediatric cases.
- Consistent levels of agreement across both zonal hospitals and health centers.

#### Collective Action

- Varying degrees of agreement on workability, interpersonal confidence, and organizational support.
- Generally consistent approach across both settings, with a notable difference in relational integration favoring the zonal hospital.
- Challenges: Inadequate supplies and lack of electricity noted as barriers to PACE implementation.

#### Reflexive Monitoring

- Strong agreement in the collective and individual appraisal of PACE.
- Consistent agreement across both settings in the appraisal and modification of PACE.
- Challenge: Inaccessibility of learned material for future reference.

#### Feasibility, Acceptability, Scalability

- PACE is generally feasible, acceptable, and potentially scalable across different healthcare settings, with some variability due to challenges and material inaccessibility.

## Discussion

### Interpretation of Findings

The mixed-methods pilot study was designed to explore the feasibility and acceptability of the PACE intervention among healthcare providers in Mwanza, Tanzania. Utilizing the Normalization Process Theory (NPT) as a guiding framework, the study revealed several key insights.

The NoMAD general questions regarding “Familiarity of PACE,” “PACE as current work,” and “PACE as future work” reveal insights into respondents’ perceptions of how well the intervention is being received in their work environment. The higher scores indicate a more positive outlook towards normalization, which can be a useful metric for stakeholders, but the wide variability of responses for familiarity and current work indicate that this optimism is not universal. PACE appears to be well-understood and recognizable among those who are implementing it, already well integrated into routine work, and respondents feel positive that PACE has the potential to become a normalized part of work in the future.

Firstly, the high levels of coherence among healthcare providers indicate that PACE is well-understood and aligns with the existing goals and practices of healthcare facilities. This is a crucial factor for the successful implementation of any healthcare intervention, as a clear understanding (i.e., how providers made sense of and accept change) among stakeholders is often the first step towards effective implementation.(27)

Secondly, the strong cognitive participation suggests that healthcare providers are not only willing but also eager to engage with PACE. The fact that providers enrolled and rallied behind PACE may indicate that the benefits were clear to them, so they were sufficiently motivated to invest their thoughts and energy into PACE, though at varying levels. This finding is in agreement with what was found by Agreli and colleagues.(28) This is particularly important in the context of pediatric care, where timely and effective interventions can have a Significant impact on patient outcomes. The willingness of healthcare providers to engage with PACE is a positive indicator of its long-term sustainability.

Thirdly, the study found varying degrees of collective action among healthcare providers. While there was general agreement on the workability and benefits of PACE, some challenges were noted, particularly in terms of resource availability and infrastructural support. However, as pointed out by MacCrorie and colleagues,(29) the extent to which the providers perceived that PACE had prepared them for implementation was influenced by the perceived compatibility of PACE within existing work practice. These challenges need to be addressed to ensure the effective and sustainable implementation of PACE.

Lastly, the study was limited in its ability to assess reflexive monitoring due to its short duration. However, the initial findings suggest that healthcare providers find value in PACE and are likely to continue using it, subject to certain improvements. Ample time is needed to allow for reflexive monitoring to mature and be realized. This is consistent with a study by Mishuris and colleagues who found that monitoring domain had the lowest scores due to being a future step in the implementation process.(30)

### Implications for Implementation Science and Pediatric Acute Care

The study’s findings have several implications for the fields of implementation science and pediatric acute care. From an implementation science perspective, the study demonstrates the utility of NPT as a conceptual framework for understanding the complexities involved in implementing adaptive e-learnings in LMICs. We observed, like studies reported by May and colleagues,(18) that coherence or sense-making was seen as a necessary precursor to participation, and a degree of cognitive participation was required before collective action-in the form of an actual implementation process-could take place. Sense-making work was also found to be a key to the successful implementation of an enhanced recovery after surgery programme.(31) The use of NPT allowed for a nuanced understanding of the various factors that could influence the implementation of PACE, providing valuable insights that could be applied to other healthcare interventions.

In the context of pediatric acute care, the study’s findings are particularly Significant. The strong agreement among healthcare providers on the benefits of PACE for managing specific pediatric cases suggests that the program could be a valuable addition to pediatric acute care at different facility types to increase provider proficiency. Given the often time-sensitive nature of pediatric cases, the effective and efficient training provided by PACE could lead to improved patient outcomes.

### Limitations

While the study provides valuable insights, it is not without limitations. The most Significant limitation is the small sample size, which affects the generalizability of the findings. Another limitation is the potential for response bias, given our response rate. Additionally, the study’s short duration did not allow for a comprehensive assessment of all NPT constructs, particularly reflexive monitoring. This limits our understanding of the long-term sustainability and impact of PACE. The study is limited by its reliance on self-reported data, which may introduce social desirability bias. However, the mixed-methods approach and methodological triangulation enhance the robustness of the findings.

### Recommendations for Future Research

Given the limitations and the scope of this pilot study, several avenues for future research are evident. Longitudinal studies are needed to assess the long-term sustainability and impact of PACE on provider proficiency, patient outcomes and quality of care. Such studies could provide insights into how PACE is embedded and integrated into routine healthcare practices over time.

Additionally, more rigorous qualitative research designs could be employed to further explore the nuances of each NPT construct. Detailed case studies and ethnographic studies could provide a more in-depth understanding of the challenges and opportunities associated with implementing PACE.

Furthermore, future research could focus on the scalability of PACE, exploring how the program could be adapted for different healthcare settings or for healthcare systems in other countries. This could include an assessment of the resource implications of scaling up PACE, as well as an evaluation of the training and support needed for effective implementation in different contexts.

## Conclusions

This study provides valuable insights into the feasibility and acceptability of the PACE program among healthcare providers in Mwanza, Tanzania. The findings suggest that PACE is well-received and aligns well with the goals of healthcare providers, particularly in the context of pediatric care. However, challenges related to resource availability and infrastructural support need to be addressed to ensure the program’s effective and sustainable implementation. The study also highlights the utility of NPT as a conceptual framework for understanding the complexities involved in implementing healthcare interventions, providing valuable insights for both implementation science and pediatric acute care.

## Data Availability

Deidentified participant data from this study are available upon reasonable request. Interested researchers may obtain the data by contacting the corresponding author, Dr. Peter Meaney, at meaneypa@stanford.edu. Access to the data will be granted following approval by an independent review committee, established to evaluate the scientific validity and ethical justification of the proposed use. Please note that only the deidentified participant data is available, and no additional supporting information, such as study protocols or statistical analysis plans, will be provided. This process ensures that the data is used responsibly and in accordance with ethical research standards.

## Abbreviations

BMC: Bugando Medical Centre
e: learning-Electronic Learning
FGD: Focus Group Discussion
IDI: In-Depth Interview
LMIC: Low-or-Middle-Income Country
NPT: Normalization Process Theory
NoMAD: Normalization Measure Development
PACE
